# Immobilization of transaminase from *Bacillus licheniformis* on copper phosphate nanoflowers and its potential application in the kinetic resolution of *RS*-α-methyl benzyl amine

**DOI:** 10.1186/s40643-021-00474-3

**Published:** 2021-12-16

**Authors:** Shraddha Lambhiya, Gopal Patel, Uttam Chand Banerjee

**Affiliations:** 1 Department of Pharmaceutical Technology (Biotechnology), National Institute of Pharmaceutical Education and Research, Sector-67, S.A.S. Nagar, 160062 Punjab India; 2 Departments of Biotechnology, Amity University, Sector 82A, IT City, International Airport Road, Mohali, 5300016 India; 3 Sagar Institute of Pharmacy and Technology, Gandhi Nagar Campus Opposite International Airport, Bhopal, 462036 MP India

**Keywords:** Transaminase, Protein purification, Hybrid nanoflowers (NFs), Nanobiocatalyst

## Abstract

This study reports the isolation and partial purification of transaminase from the wild species of *Bacillus licheniformis*. Semi-purified transaminase was immobilized on copper nanoflowers (NFs) synthesized through sonochemical method and explored it as a nanobiocatalyst. The conditions for the synthesis of transaminase NFs [TA@Cu_3_(PO_4_)_2_NF] were optimized. Synthesized NFs revealed the protein loading and activity yield—60 ± 5% and 70 ± 5%, respectively. The surface morphology of the synthesized hybrid NFs was examined by scanning electron microscopy (SEM) and transmission electron microscopy (TEM), which revealed the average size to be around 1 ± 0.5 μm. Fourier-transform infrared (FTIR) was used to confirm the presence of the enzyme inside the immobilized matrix. In addition, circular dichroism and florescence spectroscopy were also used to confirm the integrity of the secondary and tertiary structures of the protein in the immobilized material. The transaminase hybrid NFs exhibited enhanced kinetic properties and stability over the free enzyme and revealed high reusability. Furthermore, the potential application of the immobilized transaminase hybrid NFs was demonstrated in the resolution of racemic α-methyl benzylamine.

**Graphical Abstract:**

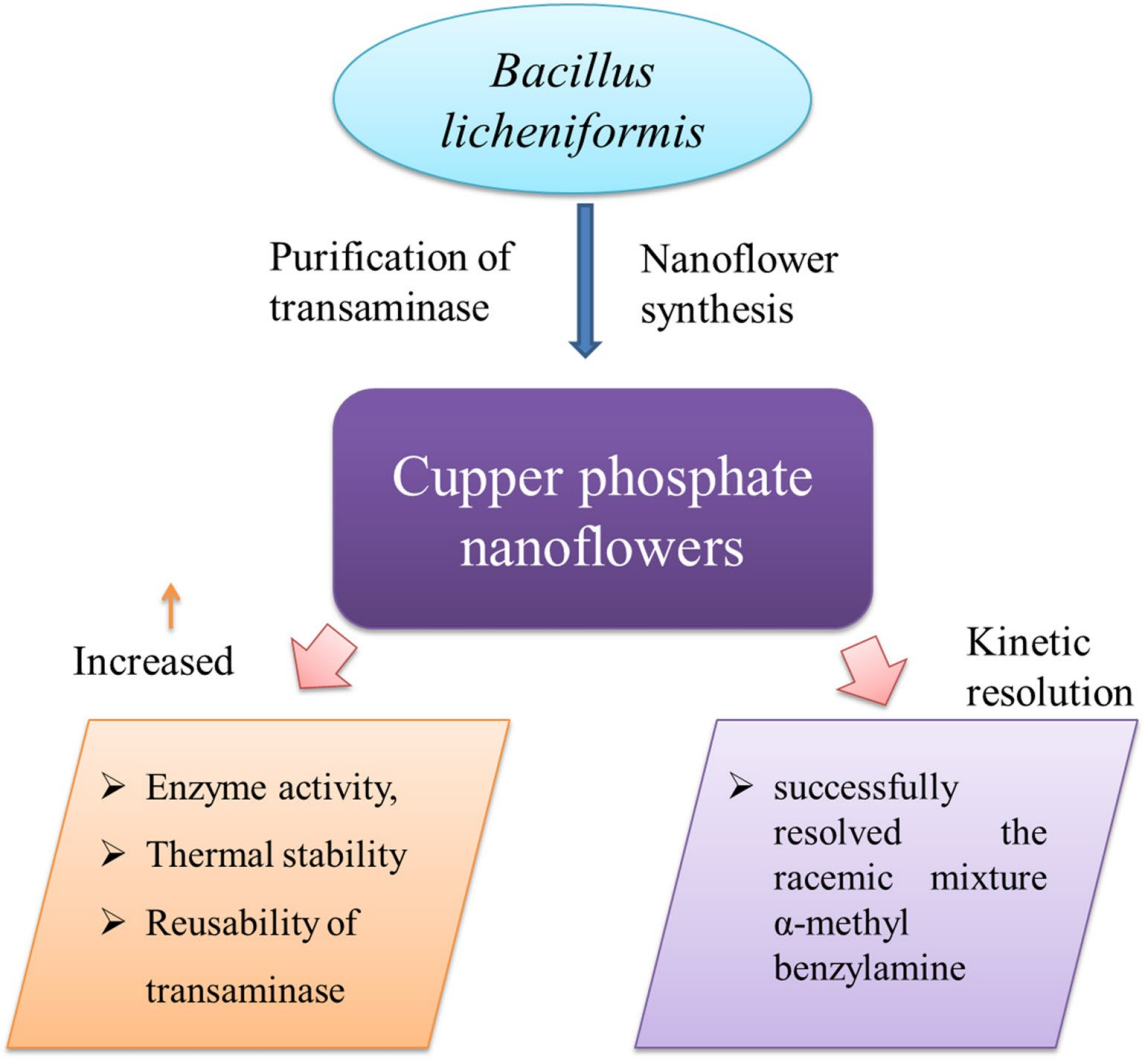

## Introduction

Biocatalysts are extensively used in biotransformation applications, particularly in the environmental and industrial segments. The cell-free biocatalysts are recognized and expected to be used more in biotransformation reactions, because they have greater specificity with regard to substrate and reaction rate, a higher tolerance towards higher substrate concentration, and are appropriate for separation of the product (Rollin et al. 2013). Enantiomerically pure amines and *α*/*β*-amino acids play a crucial role in living organisms, and also play an important role in agrochemical, chemical, and pharmaceutical industries, as intermediate or final products (Schätzle et al. 2011). Thus, synthesis of achiral amine compound is an efficient and cost-effective approach in the biocatalyst reaction, and is an attractive alternative for the conventional chemical methods (Paetzold and Bäckvall 2005). Among the different biocatalysts, transaminase (TA) has recently been received great attention as a promising catalyst, due to its ability to produce a wide range of optically pure amines and unnatural amino acids (Schätzle et al. 2011; Mathew and Yun 2012; Shin et al. 2013, 2015; Päiviö and Kanerva 2013; Paul et al. 2014). Transaminases (TAs) catalyse the transfer of an amino group from the amino donor to the acceptor, employing the approach of kinetic resolution or asymmetric synthesis (Höhne and Bornscheuer 2012; Nestl et al. 2014). The amino group transfer is mediated by a vitamin B6-based cofactor pyridoxal 5′-phosphate (PLP), reversibly bound to a catalytic lysine of the enzyme, via an imine bond, which assists the reaction by acting as a transient “custodian” of the amino group (Höhne and Bornscheuer 2012; Homaei et al. 2013; Guo and Berglund 2017).The extensive industrial applications and desirable characteristics of the biocatalyst are often hindered by operation stability, such as, easy degradation of their molecular structure (at higher temperatures, at acidic or basic pH, in the presence of organic solvents, and in long-term storage) and their cumbersome recovery and re-use, which strictly limits their use. In recent times, numerous immobilization techniques have been used to overcome these problems (Homaei et al. 2013; Ahmad and Sardar 2015). At present various efficient methods are being used for immobilization of enzymes, such as, adsorption, covalent binding, entrapment, and cross-linking (Lei et al. 2006; Sheldon 2007; Brady and Jordaan 2009; Wang et al. 2014; Altinkaynak et al. 2016). Nanobiocatalysis is an emerging innovation that synergistically fuses nanotechnology with biocatalysts, and has more advantages, for example, large surface area, mass ratios, control over the size on a nanometer-scale, with a broad range of functionalities, and other attractive electronic, optical, magnetic, and catalytic properties (Kim et al. 2008; Jariwala et al. 2013; Misson et al. 2015; Lin et al. 2016a; Mansouri et al. 2017; Pakapongpan and Poo-arporn 2017). Within the past few decades various novel approaches, such as, single enzyme nanoparticles, metal–organic frameworks(Chen et al. 2017; Liu et al. 2021; Luan et al. 2021), silica nanocarriers (Du et al. 2013), polymer nanocarriers (Lin et al. 2012), cross-linked enzyme aggregates (Kartal 2016; Care et al. 2017), and enzyme nanocarrier fabricated hybrid organic–inorganic nanostructures (Kharisov 2008) have been reported for structural and functional modification of enzymes. Hybrid organic–inorganic NFs is a promising breakthrough in enzyme immobilization, which exhibits enhanced enzymatic activity and stability as compared to free enzymes, which may be attributed to the confinement of the enzyme in the core of the NFs and high-surface area (Ge et al. 2012; Lee et al. 2015; Li et al. 2021). The combined functionalities of the protein and the inorganic material of the hybrid NFs, enables its application in biosensors (Gao et al. 2020; Zhu et al. 2017, 2018), biofuel cells (Maleki et al. 2019), and in biocatalysis (Rai et al. 2018).The most peculiar facet of the protein–inorganic hybrid NFs is synthesized by the conventional incubation method (reaction of mixture after three days, at room temperature), which significantly reduces their use in actual application. Nanoflower synthesis through the ultrafast sonochemical method accomplishes the limitation of the conventional method (Batule et al. 2015; Dwivedee et al. 2018).

The present study reports the isolation and purification of transaminase (EC 2.6.1.B16) from *Bacillus licheniformis*, along with its hybrid enzyme–inorganic nanoflower synthesis [TA@Cu_3_(PO_4_)_2_NF]. The transaminase hybrid nanoflower morphology and enzyme activity have been modulated by the duration of ultrasonic treatment, sonication power, enzyme/metal salt concentration, and buffer pH. The surface morphology of hybrid NFs has been characterized by scanning electron microscopy (SEM), transmission electron microscopy (TEM), Fourier-transform infrared (FTIR), circular dichroism (CD), and florescence spectroscopy. The transaminase hybrid NFs exhibit enhanced kinetic properties and stability over the free enzyme and reveal high reusability. Furthermore, the potential application of the immobilized transaminase hybrid NFs has been demonstrated in the resolution of racemic α-methyl benzylamine.

## Materials and method

### Materials

*Bacillus licheniformis* MTCC 429 was procured from Institute of Microbial Technology, Chandigarh, India. CaCl_2_.2H_2_O, CoCl_2_.6H_2_O, CuSO_4_.5H_2_O, MnSO_4_.H_2_O, benzyl amine, (*S*)-*α*-methyl benzyl amine (*S-α*-MBA), (RS)-α-methyl benzyl amine, pyruvic acid, pyridoxal-5′-phosphate (PLP), phenyl methyl sulfonyl fluoride (PMSF), and methyl tertiary butyl ether (MTBE) were purchased from Sigma Aldrich. Yeast extract, meat extract, K_2_HPO_4_, MgSO_4_, glutamic acid were obtained from HiMedia.

### Production and purification of transaminase

An inoculum (2% v/v) of *B. licheniformis* was grown in the production medium containing galactose (5 g/L), yeast extract (15 g/L), meat extract (15 g/L), K_2_HPO_4_ (4 g/L), MgSO_4_ (0.2 g/L), glutamic acid (1 g/L); pH was adjusted to 6. The fermentation was carried out at 37 °C for 28 h and 150 RPM. At the end of fermentation, the cells were harvested by centrifugation (7000 RPM for 20 min at 4 °C) and washed three times with 50 mM Tris–HCl buffer (8 pH).

The wet cells (2 g) were suspended in 10 mL 50 mM Tris–HCl buffer (8 pH) containing 20 µM PLP and 1.0 mM PMSF. Subsequently the cells were disrupted by probe sonication for 10 min at 4 °C. The sonicated cell suspension was centrifuged at 10,000 RPM for 30 min at 4 °C and cell-free lysate (crude enzyme solution) was collected and stored at 4 °C. Afterward transaminase was partially purified using 50 mM Tris buffer (pH 8) containing 10 µM PLP pre-equilibrated Macro-Prep High strong anion exchange column (Patil et al. 2017b). The cell-free extract was loaded onto a column and unbound protein washed with same buffer until no protein was found. Protein was eluted with a gradient of NaCl from 0.075 M, 0.1 M, 0.15 M, 0.2 M, 0.25 M, 0.3 M, 0.4 M to 1 min 50 mM Tris buffer (pH 8). The protein elution pattern was determined by Bradford assay (Bradford 1976) and elution of transaminase was measured through transaminase activity assay for all the collected fraction. Purification profile of enzymes was confirmed by reducing SDS PAGE (12% polyacrylamide gel) and Coomassie blue staining. The transaminase active fractions were collected, concentrated and washed with phosphate-buffered saline (pH 7.4) thrice using MILLIPORE^®^ centricon tube (30,000 MWCO) (Patil et al. 2017a).

### Activity measurement

Free and immobilized transaminase activity were measured by copper sulphate methanol assay (Hwang and Kim 2004). The staining solution was prepared by mixing 300 mg copper sulphate in 0.5 mL water followed by the addition of 30 mL methanol. For the assay benzyl amine (200 µL, 200 mM) was used as an amino donor and pyruvic acid (200 µL, 100 mM) was used as an amino acceptor in presence of pyridoxal 5′-phosphate (100 µL, 0.5 mM) as a cofactor of transaminase in phosphate buffer (300 µL, 50 mM, and pH 7). Enzyme solution (200 µL) was added in the above solution and incubated at 37 °C for 10 min. Reaction mixture was then cooled at room temperature for 10 min and 200 µL staining solution was added. It was further centrifuged to remove precipitate and UV absorbance of blue colour supernatant was measured at 650 nm. One unit (U) of transaminase is expressed as the amount of enzyme that releases 1 µM L-alanine per minute under optimal assay conditions (Du et al. 2013). In this reaction, alanine is formed which gives a blue complex with the Cu^2+^ ion and maximum absorbance at 650 nm.

### Synthesis of copper phosphate nanoflower with transaminase [TA@Cu_3_(PO_4_)_2_NF]

The transaminase hybrid nanoflowers were synthesized by mixing 3 mL phosphate-buffer saline (pH 7.4) containing 0.25 mg/mL enzyme with 20 µL CuSO_4_.5H_2_O in water (120 mM), mixed vigorously using vortex mixer. The mixture was then sonicated for definite time period in bath sonicator (power-sonic 505) at room temperature and 40 kHz frequency. After sonication, transaminase hybrid nanoflowers were centrifuged (3500 RPM, 20 min, and 4 °C) and washed twice with phosphate-buffered saline (pH 7.4). The enzyme formed a complex with copper ions, which built a nucleation site for the growth of primary crystals of copper phosphate. The interaction of transaminase with copper ions leads to the growth of flower-like particles that have nanoscale structures (Ge et al. 2012). Previous studies also revealed that this type of hybrid nanoflowers improved enzyme stability and activity as compared to free enzyme (Lee et al. 2015; Zhao et al. 2021).

### Optimization of immobilization parameters for the synthesis of TA@Cu_3_(PO_4_)_2_NF

#### Effect of ultrasonic treatment time

The time of ultrasonic treatment greatly influences the appropriate formation of nanoflower and encapsulation of enzyme (Soni et al. 2018). Here we studied the effect of sonication time (5, 10, 15, 20, 25 and 30 min) on NFs synthesis by sonicating the reaction mixture of phosphate-buffer saline (pH 7.4) containing 0.25 mg/mL enzyme with 20 µL CuSO_4_.5H_2_O (120 mM), in a bath sonicator. The other conditions were kept constant as following: sonication power medium (170 W), pH 7.4 of phosphate-buffer saline and treatment frequency 40 kHz.

#### Screening of metal salts

Salts played an important role in the synthesis of NFs. Initially specified metal salts (CaCl_2_.2H_2_O, CoCl_2_.6H_2_O, CuSO_4_.5H_2_O and MnSO_4_.H_2_O) were screened individually at a concentration of 120 mM for the synthesis of NFs with the maximum transaminase entrapment, optimum size and shape (Dwivedee et al. 2018). The other conditions were kept constant as following: sonication power medium (170 W), pH 7.4 of phosphate-buffer saline containing 0.25 mg/mL enzyme, and treatment time 20 min.

#### Effect of ultra-sonication power

Power of sonication is also one of the main factors which played a significant role in the synthesis of nanoflower; sometime small power is not enough to initiate the NFs synthesis and higher power may disturb the shape and size of nanoflower (Dwivedee et al. 2018). So the effects of different sonication power [high (200 W), medium (170 W) and low 140 (W)] were also studied individually on the synthesis of nanoflower in a reaction solution comprising enzyme (0.25 mg/mL in PBS, pH 7.4) and metal salt (CuSO_4_.5H_2_O, 120 mM) sonicated for 20 min in bath sonicator.

#### Optimization of enzyme/salt concentration ratio

Furthermore, the effect of enzyme and salt concentration on NFs formation were studied by sonicating 3 mL PBS (pH7.4) comprising different concentrations of enzyme (0.2 mg/mL, 0.25 mg/mL, and 0.3 mg/mL) and copper salt (0.66 mM, 0.8 mM, and 1 mM final concentration) at optimized sonication power (170 W) and treatment time (20 min).

#### Optimization of buffer pH

Various studies had shown that buffer pH plays a crucial role in the NFs synthesis and it influences the size and texture of nanoflower (Jung et al. 2009; Luo et al. 2017). Hence, size of NFs is controlled by varying buffer pH. The effect of pH in NFs synthesis was studied at different pH’s (3.4, 5.5, 7.4, 8, and 9) keeping all other optimized conditions constant.

### Immobilization efficiency

Transaminase hybrid nanoflowers [TA@Cu_3_(PO_4_)_2_NF] were studied for their capacity to immobilize the enzyme and it was evaluated through the transaminase activity, specific activity, protein loading, yield and activity yield of the immobilized enzyme (Neto et al. 2015).

$${\text{Specific}} \; \text{activity}\; (\text{U}/\text{mg} \;\text{protein}) = \frac{{\text{Activity}\; \text{of} \;\text{immobilized} \; \text{transaminase}}} {{\text{Amount}\, \text{of} \;\text{protein}\, \text{loading}}},$$

$${\text{Protein}} {\text{loading}}\; {\text{yield }}\left( \% \right) = \frac{{{\text{Amount}}\; {\text{of}} \;{\text{protein}}\, {\text{loading}}}}{{{\text{Amount}} \;{\text{of}}\; {\text{protein}}\; {\text{introduced}}}} \times 100\% ,$$

$${\text{Activity}} {\text{yield }}\left( \% \right) = \frac{{{\text{Specific}} \;{\text{activity}}\; {\text{of}}\, {\text{immobilized}}\; {\text{transaminase}}}}{{{\text{Specific}}\; {\text{activity}} \;{\text{of}}\; {\text{free}}\; {\text{transaminase}}}} \times 100\% .$$

### Characterization of transaminase nanoflower

The surface morphology of hybrid nanoflowers has been characterized by scanning electron microscopy (SEM, Hitachi S3400N) and transmission electron microscopy (TEM, FEI Tecnai™). Fourier-transfer infrared (FTIR) spectroscopy was used to examine functional groups of chemical compounds; spectra were collected from Perkin Elmer FTIR spectrometer with ATR synthesis monitoring system in 4000–650 cm^−1^ infrared region. The secondary structure changes in enzyme was investigated using circular dichroism (CD) spectroscopy, CD spectra were recorded on a JASCO J-810 CDD instrument at 25 °C. Spectra manager software was used to analyse the protein secondary structures fraction ratio. The change in tertiary structure was determined by using fluorescence spectroscopy (Perkin Elmer, LS-50B). The fluorescence spectra were scanned at 300–450 nm emission range at 280 nm excitation wavelength (*λ*_ex_) (Soni et al. 2018; Dwivedee et al. 2018).

### Application of TA@Cu_3_(PO_4_)_2_NF in the resolution of (RS)-α-methyl benzyl amine

The reusability of hybrid transaminase nanoflowers was studied for the kinetic resolution of (*RS*)-α-methyl benzyl amine. The reaction was carried out with 300 µL α-methyl benzyl amine (500 mM) as amino donor, 200 µL pyruvic acid (100 mM) as amino acceptor, 200 µL transaminase hybrid nanoflowers solution (0.5 mg/mL enzyme concentration), 100 µL PLP (0.5 mM) as a cofactor of the enzyme and 200 µL sodium phosphate buffer (50 mM, pH 7.4) at 37 °C and 150 RPM. The reaction was terminated after 6 h; subsequently it was centrifuged (3500 RPM, 20 min, and 4 °C) and collected the nanoflowers. From the supernatant residual α-methyl benzyl amine was extracted in MTBE, dried, suspended in isopropanol and analysed using chiral HPLC (Chiralcel OD-H column, 0.5 mL/min flow rate, hexane:2-propanol::90:10, 210 nm UV detection, and 25 °C column temperature). Collected TA@Cu_3_(PO_4_)_2_NF were washed with phosphate buffer (50 mM, pH 7.4) and used in the successive cycle. The initial activity of enzyme was considered as 100% kinetic resolution of *(RS*)-α-methyl benzyl amine.

## Results and discussion

### Purification of transaminase

**Fig. 1:**
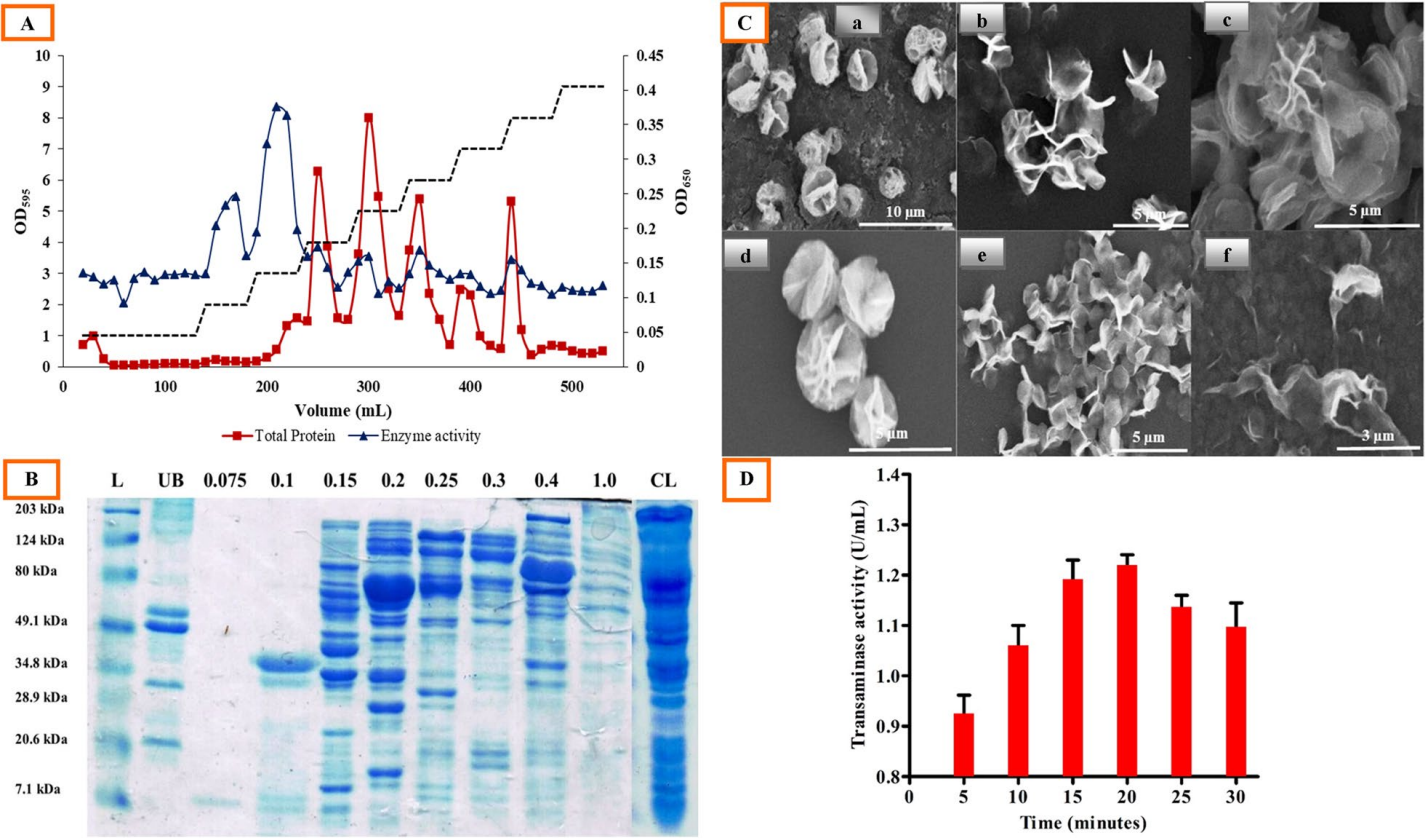
**A** Chromatogram of transaminase purification in anion exchange column; **B** SDS-PAGE analysis of different elution fractions of anion exchange chromatography*; **C** SEM images of sonication time optimization study on nanoflower synthesis, **a** 5 min, **b** 10 min, **c** 15 min, **d** 20 min, **e** 25 min, and **f** 30 min; **D** TA activity of hNFs synthesized at different sonication time intervals. *Proteins were separated on a 12% polyacrylamide gel in the presence of 1% SDS. Here, lane 1 represents marker proteins (molecular mass 203, 124, 80, 49.1, 34.8, 28.9, 20.6, 7.1 kDa), lane 2 depicts unbound fraction, lane 3 depicts 0.075 M NaCl elution fraction, lane 4 depicts 0.1 M NaCl elution fraction, lane 5 depicts 0.15 M NaCl elution fraction, lane 6 depicts 0.2 M NaCl elution fraction, lane 7 depicts 0.25 M NaCl elution fraction, lane 8 depicts 0.3 M NaCl elution fraction, lane 9 depicts 0.4 M NaCl elution fraction, lane 10 depicts 1.0 M NaCl elution fraction, and lane 11 depicts cell lysate

The total protein was measured in all the eluted fractions using the Bradford assay, which depicted a protein elution pattern (Fig. 1A). The specific activity of the partially purified transaminase was increased from 0.31 U/mg (cell-free extract) to 9.03 U/mg (Macro-Prep High Q active fraction). Partial purification of the transaminase yielded a 29-fold purification of the transaminase with a single step. The representative results of the purification procedure are given in Table 1. The significant increase in TA activity obtained using the Macro-Prep High Q protein purification step could be a result of the removal of an inhibitory substance in the cell-free extract. Appearance of a prominent band, around 40 kDa, in a 0.1 M NaCl elution fraction, with the highest transaminase activity (13 U/mL), demonstrated the presence of a partially purified transaminase in this fraction (Fig. 1B).

**Table 1:** Purification scheme of TA from *B. licheniformis*

Purification step	Total protein (mg)	TA activity (U/mL)	Specific activity (U/mg)	Fold purification	Yield (%)
Cell-free extract	24.07	7.52	0.31	1	100.00
Macro-prep high Q	1.50	13.54	9.03	29	180.05

### Synthesis of transaminase nanoflowers [TA@Cu_3_(PO_4_)_2_NF]

Synthesis of hybrid NFs through sonication hastened the process as compared to the conventional incubation method. Sonochemical synthesis of the nanoflower process included three steps: (i) nucleation and formation of primary crystals; (ii) growth of the crystals, and (iii) formation of a nanoflower assembly, by incorporation of metal salts and proteins (Dwivedee et al. 2018; Ge et al. 2012; Lee et al. 2015). To synthesize the protein–inorganic hybrid NFs through sonication, an aqueous PBS solution containing copper (II) sulfate and enzyme was sonicated for a definite time, followed by centrifugation at 3500 RPM for 20 min, when it obtained blue colour precipitates. Sonication parameters, such as, sonication time, ultra-sonication power, pH of the buffer, and enzyme–metal salt concentration were optimized, to obtain robust and effective transaminase NFs. Ultrasonication treatment is an innovative method of nanoflower synthesis which considerably decreases the synthesis time. As conventional methods were taken 3 days for completions of a reaction while the same reaction was completed within 10–20 min by ultrasound treatment (Chung et al. 2018). Here the sonication method might have permitted the copper phosphate to rapidly complete self-assembly progression by consistently providing high energy to the structure (Ge et al. 2012; Lee et al. 2015; Zhao et al. 2021).

#### Effect of ultrasonic treatment time

A reaction mixture containing 0.25 mg/mL enzyme with 20 µL CuSO_4_.5H_2_O (120 mM) in a bath sonicator was investigated to find out the effect of sonication time. The surface morphology and growth steps of the nanoflower, at different time intervals, were observed through SEM (Fig. 1C). Sonication of the reaction mixture for 5 min resulted in spherical precipitates of enzyme–metal ion combinations (Fig. 1Ca). At this early stage of growth, only few proteins (transaminase) formed complexes with Cu^+2^, predominantly through the coordination of amide groups in the protein backbone (Ge et al. 2012; Hua et al. 2016; Lin et al. 2016b). These complexes provided a location for nucleation of the primary crystal. The first time petal formation was observed at 10 min sonication of the reaction mixture due to successive growth of protein–Cu^+2^ crystals into large agglomerates (Fig. 1Cb). The nanoflower formation through the anisotropic growth of protein nanopetals aggregates and primary crystals was observed at 15 min of sonication (Fig. 1Cc). Further sonication of the reaction mixture up to 20 min displayed blooming flowers (Fig. 1Cd). The assembling steps for NFs are mentioned herewith: protein induces the nucleation of the Cu_3_(PO_4_)_2_ crystals to form the scaffold for the petals and serve as ‘glue’ to bind the petals together (Ge et al. 2012; Lin et al. 2016b). Prolonged sonication for 25 min leads to distortion of the nanoflower assembly into individual petals (Fig. 1Ce). Sonication of the reaction mixture beyond 25 min resulted in complete disruption of the nanopetal morphology (Fig. 1Cf). In addition to the morphological study, transaminase activity was also observed in different time-sonicated samples. Figure 1D clearly reveals that enzyme activity increased continuously from 5 to 20 min and it was maximum in the 20-min sonicated sample, where the NFs are completely formed. After 20 min of treatment, the enzyme activity again decreased, might be due to distortion of the nanoflower assembly into individual petals, as shown in Fig. 1Ce, f (Wang et al. 2014). Based on the observation of nanoflower morphology and the enzyme activity of NFs prepared at different sonication times, 20 min was chosen as the optimum time for nanoflower synthesis.

#### Screening of metal salts

**Fig. 2:**
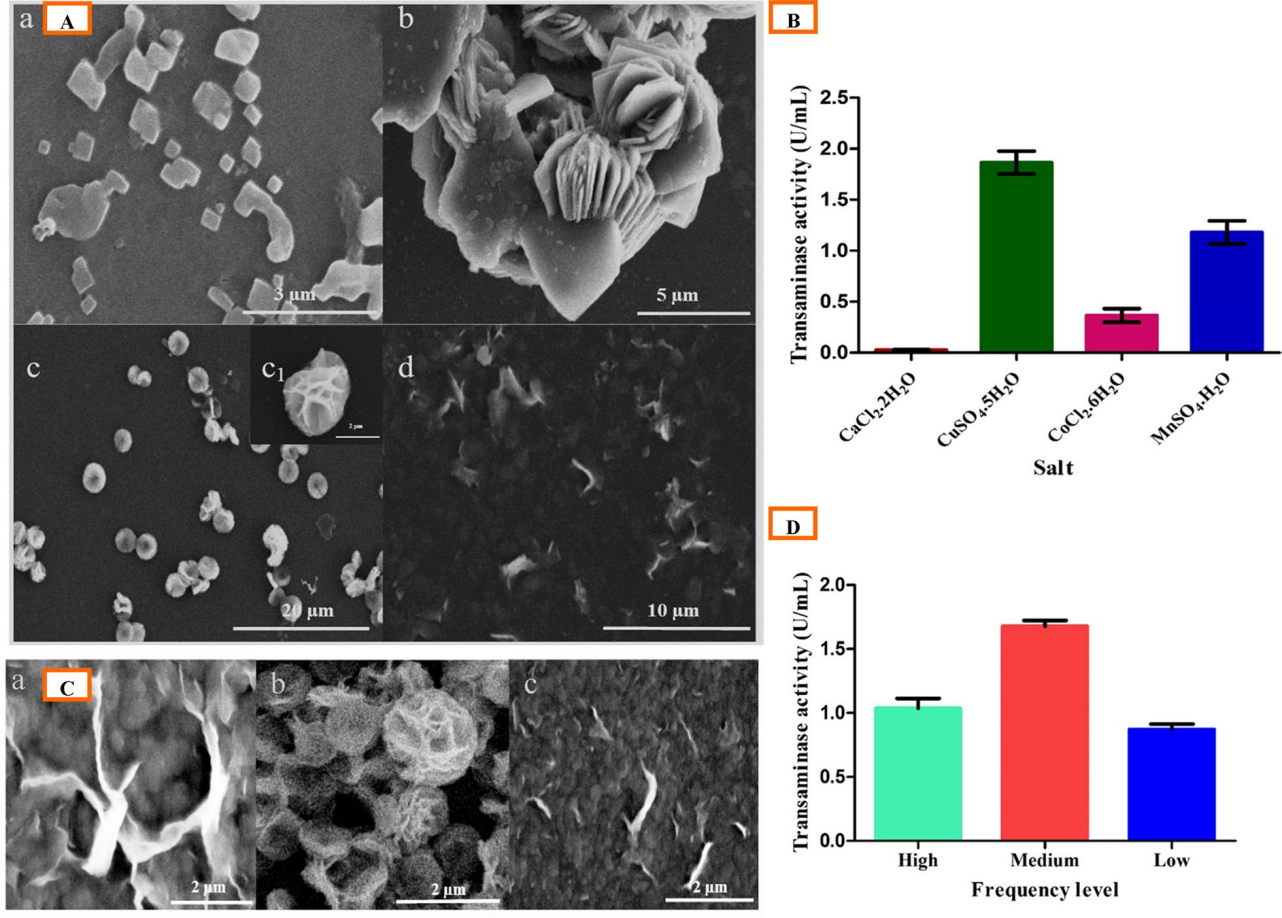
**A** SEM images of sonication-mediated transaminase nanoflower synthesis with different metal salt in 120 mM concentration, **a** CaCl_2_.2H_2_O, **b** CoCl_2_.6H_2_O, **c** CuSO_4_.5H_2_O, **d** MnSO_4_.H_2_O, and **c**_**1**_ magnified image of **c.** **B** TA activity of hybrid nanoflower synthesis with different metal salts. **C** SEM images of hybrid nanoflowers synthesized at different power level, **a** low, **b** medium, and **c** high. **D** TA activity of nanoflower synthesis at different power level

The morphology of organic–inorganic hybrid nanomaterials and their activities solely depend on the type of metal salts (inorganic components) and their complex formations with the protein (organic substance) (Dwivedee et al. 2018). Different metal salts (CaCl_2_.2H_2_O, CoCl_2_.6H_2_O, CuSO_4_.5H_2_O, and MnSO_4_.H_2_O) were evaluated individually for nanomaterial formation and their effect on the TA activity (Fig. 2A, B). Sonication of the reaction mixture with CaCl_2_.2H_2_O (Fig. 2Aa) and MnSO_4_.H_2_O (Fig. 2Ad) showed no nanoflower formation, butMnSO_4_.H_2_O precipitates had enzyme activity (Fig. 2B). CoCl_2_.6H_2_O (Fig. 2Ab) and CuSO_4_.5H_2_O (Fig. 2Ac) metal salts formed a flower-like morphology but only the CuSO_4_.5H_2_O NFs exhibited higher enzyme activity and a uniform assembly of NFs (Fig. 2A, B). The results clearly revealed that the CuSO_4_.5H_2_O was the most appropriate salt for the synthesis of NFs, with the highest transaminase activity, and these results were consistent with the previous studies on nanoflower synthesis (Ge et al. 2012).

#### Effect of the power of ultra-sonication

Sonication of a reaction mixture comprising 120 mM copper(II) sulfate and 0.25 mg/mL enzyme was performed in an ordinary bath sonicator (frequency 40 kHz) at different sonic power levels (low-1, medium-2, and high-3) for 20 min (Fig. 2C). The SEM analysis of the samples depicted that a lower sonication power (140 W) was not sufficient for nanoflower formation (Fig. 2Ca) and higher sonication power (200 W) showed distortion of the nanoflower assembly (Fig. 2Cc), whereas, the medium sonication power (170 W) resulted in the formation of well-structured NFs (Fig. 2Cb). Figure 2D exhibits the enzyme activity of different sonication power levels, where 170 W (medium) showed the maximum enzyme activity as compared to the others. These results were in consistent with the earlier studies on nanoflower synthesis (Dwivedee et al. 2018).

#### Optimization of the enzyme/salt concentration ratio

**Fig. 3:**
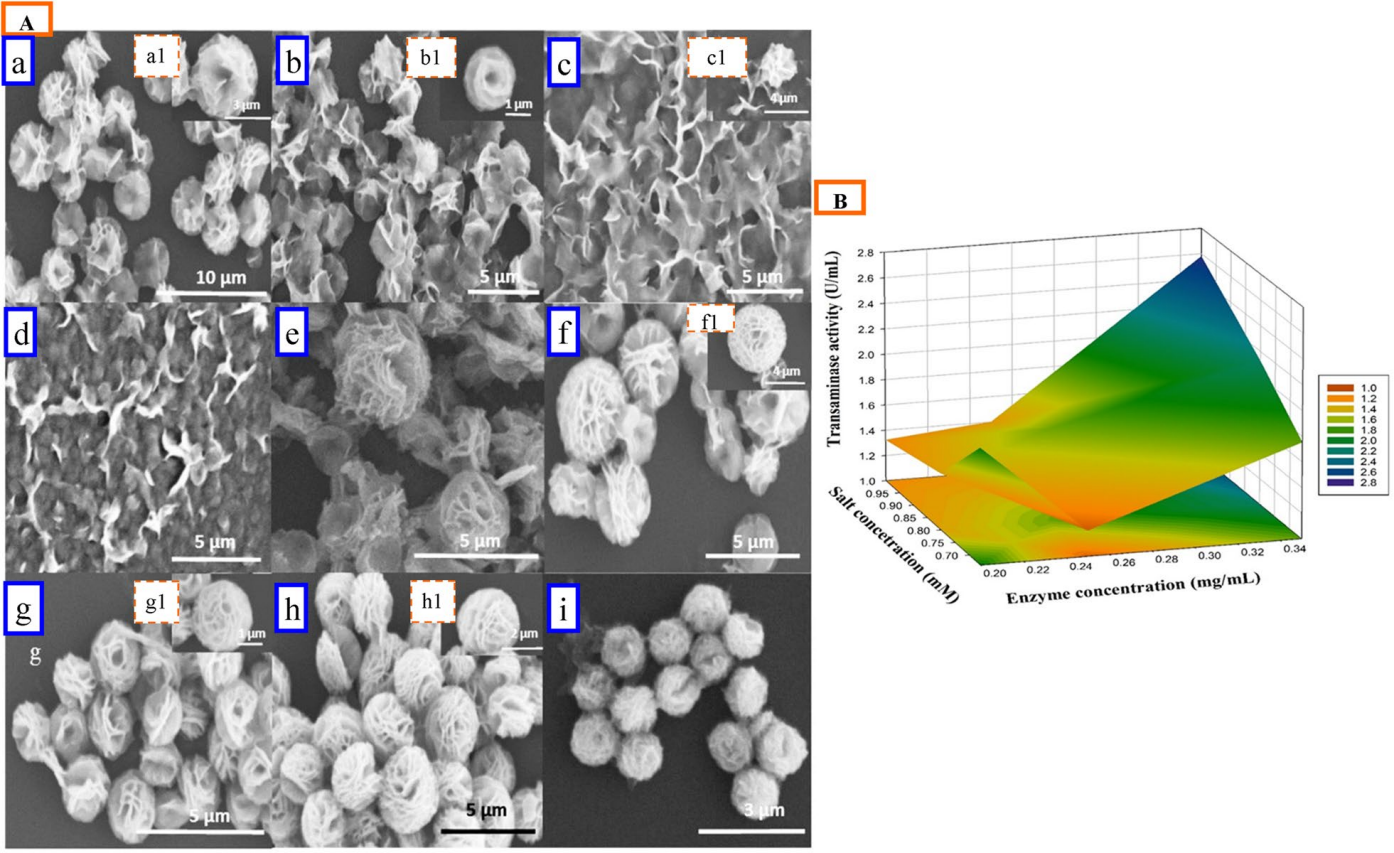
**A** SEM images of hybrid nanoflower [TA@Cu_3_(PO_4_)_2_NF] synthesis with various enzyme (mg/mL)/salt (mM) concentration, **a** 0.2/0.66, **b** 0.2/0.8, **c** 0.2/1.0, **d** 0.25/0.66, **e** 0.25/0.8, **f** 0.25/1.0, **g** 0.35/0.66, **h** 0.35/0.8, **i** 0.35/1.0, **a**_**1**_ magnified image of **a**, **b**_**1**_ magnified image **b**, **c**_**1**_ magnified image of **c**, **f**_**1**_ magnified image of **f**, **g**_**1**_ magnified image of **g**, and **h**_**1**_ magnified image of **h.** **B** TA activity of TA@Cu_3_(PO_4_)_2_NF with different enzyme/salt concentration

The effect of different concentrations of metal salts and enzymes on the morphology of transaminase NFs and enzyme activity is depicted in Fig. 3A, B, respectively. The morphology of the NFs was observed to vary distinctly between 0.9 and 6 µm under the reaction conditions (Fig. 3A). The surface morphology in SEM analysis was observed that low enzyme concentration (0.2 mg/mL) with low metal salt concentration (0.66 mM) formed nanoflowers (Fig. 3Aa). The nitrogen atom in the amide group of the protein backbone, forms complexes with Cu^+2^. Nucleation and growth of the primary crystal originates at these sites to form a separate petal (Ge et al. 2012). An unorganized large petal, without clear NFs, similar to the morphology obtained at a low enzyme concentration (0.2 mg/mL) and high salt concentration (1.0 mM) was observed, presumably revealing a lower nucleation site for nanoflower growth at a high salt concentration (Fig. 3Ac). Moderate enzyme concentration (0.25 mg/mL) formed good nanoflower arrangement with higher salt concentration (1.0 mM) (Fig. 3Af), but moderate metal salt (0.8 mM) (Fig. 3Ae), showed highest enzyme activity. A higher enzyme concentration (0.35 mg/mL) with increasing metal salt concentration demonstrated a decrease in nanoflower size, from 3 to 1 µm in diameter. A higher enzyme concentration (0.35 mg/mL) with higher metal salt [1.0 mM copper (II) sulphate] resulted in small-sized NFs, with higher enzyme activity and protein loading (Fig. 3Ai). Therefore, a 0.35 mg/mL enzyme loading and 1.0 mM metal salt concetration were optimized for the synthesis of hybrid NFs. Interactive effect of enzyme and salt concentrations have been shown in a 3D plot (Fig. 3B), which revealed a higher transaminase activity with higher concentrations of both.

#### Optimization of buffer pH

**Fig. 4:**
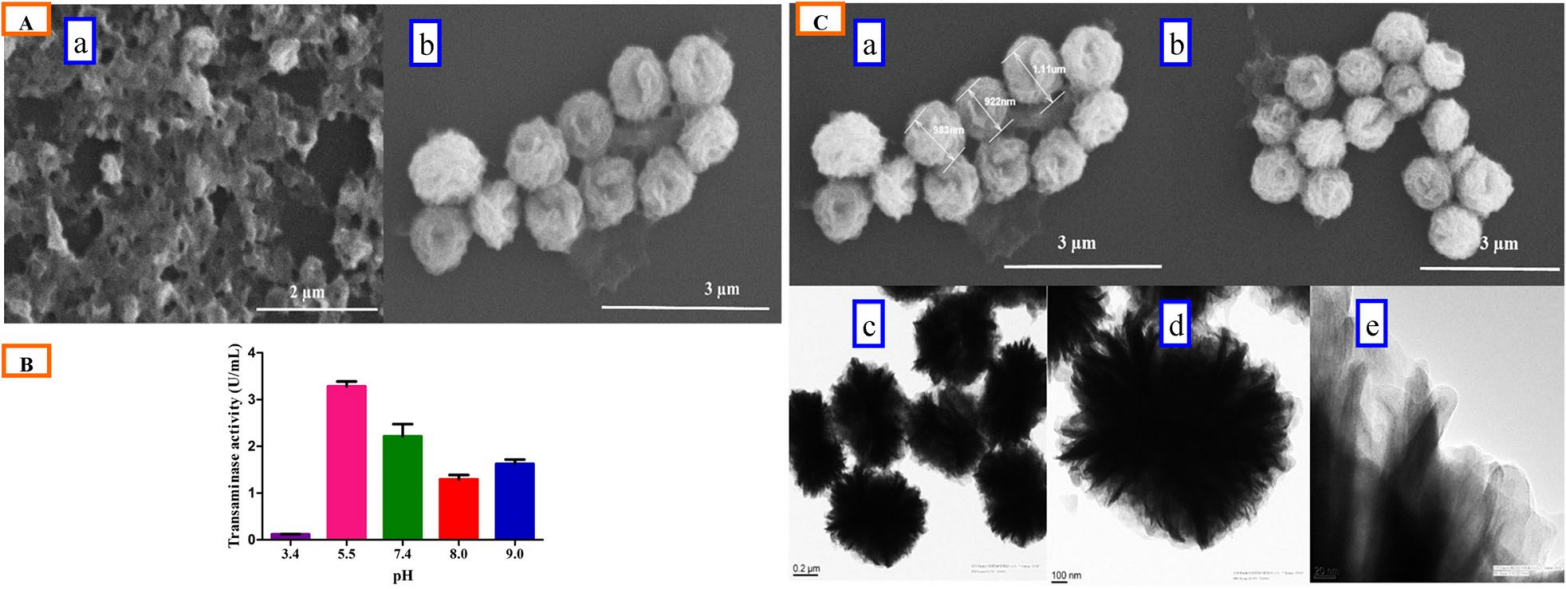
**A** SEM images of transaminase hybrid nanoflowers synthesized at, **a** pH 5.5, **b** pH 7.4.** B** TA activity of TA@Cu_3_(PO_4_)_2_NF synthesis at different pH. **C **Characterization of hybrid NFs synthesized under optimum condition, **a**, **b** SEM images of TA@Cu_3_(PO_4_)_2_NF, **c** TEM image of TA@Cu_3_(PO_4_)_2_NF, **d** magnified TEM image of **c**, **e** magnified TEM image of **d**

The effect of pH (ranging from pH 3.4 to 9.0) on nanoflower synthesis is shown in Fig. 4A, B. The nanoflower formation had not occurred in the acidic pH (3.4) (which is clearly evident in Fig. 4B) due to the negligible enzyme activity that was observed. Increasing the pH to 5.5 led to a drastic increase in the TA activity (3.29 U/mL, Fig. 4B). However, on examining the morphology of the NFs, the reaction mixture at pH 5.5 displayed a poor morphology of NFs (Fig. 4Aa), probably because the H_2_PO_4_^−^, a major anion of the acidic condition, was not easily accessible to convert the Cu_3_(PO_4_)_2_ crystal for nanoflower synthesis, compared to the neutral or alkaline pH, which had HPO_4_^−^, a major anion (Jung et al. 2009; Luo et al. 2017). However, a significant improvement in the formation of NFs had occurred at pH 7.4 (Fig. 4Ab), although the TA activity was lower (2.22 U/mL) when compared with that of at pH 5.5 (Fig. 4B). A further increase in pH, to 8 and 9, led to a reduction in the TA activity. Thus, a buffer pH 7.4 was used as the optimized pH in subsequent experiments.

### Characterization of TA@Cu_3_(PO_4_)_2_NF

Based on the above conditions, the optimum values for the sonication-mediated synthesis of TA@Cu3(PO_4_)_2_NF were as follows: duration of ultra-sonication treatment 20 min; CuSO_4_.5H_2_O as a metal salt; sonication power 170 W (medium level); buffer pH 7.4; 150 mM Copper (II) sulfate, and enzyme 0.35 mg/mL.TA@Cu_3_(PO_4_)_2_NF synthesized under the optimum condition revealed a protein loading of 60 ± 5% and activity yield of 70 ± 5%. The SEM image of TA@Cu_3_(PO_4_)_2_NF clearly revealed a flower-like structure (Fig. 4Ca, b), with average size of 1 ± 0.5 μm. However, the average size of NFs in the TEM analysis was comparatively low, around 0.7 µm (Fig. 4Cc). The TEM image of Fig. 4Cd, e showed multiple petals, which confirmed the synthesis of NFs by the aggregation of nanosized petals.

#### Fourier-transform infrared spectroscopy

**Fig. 5:**
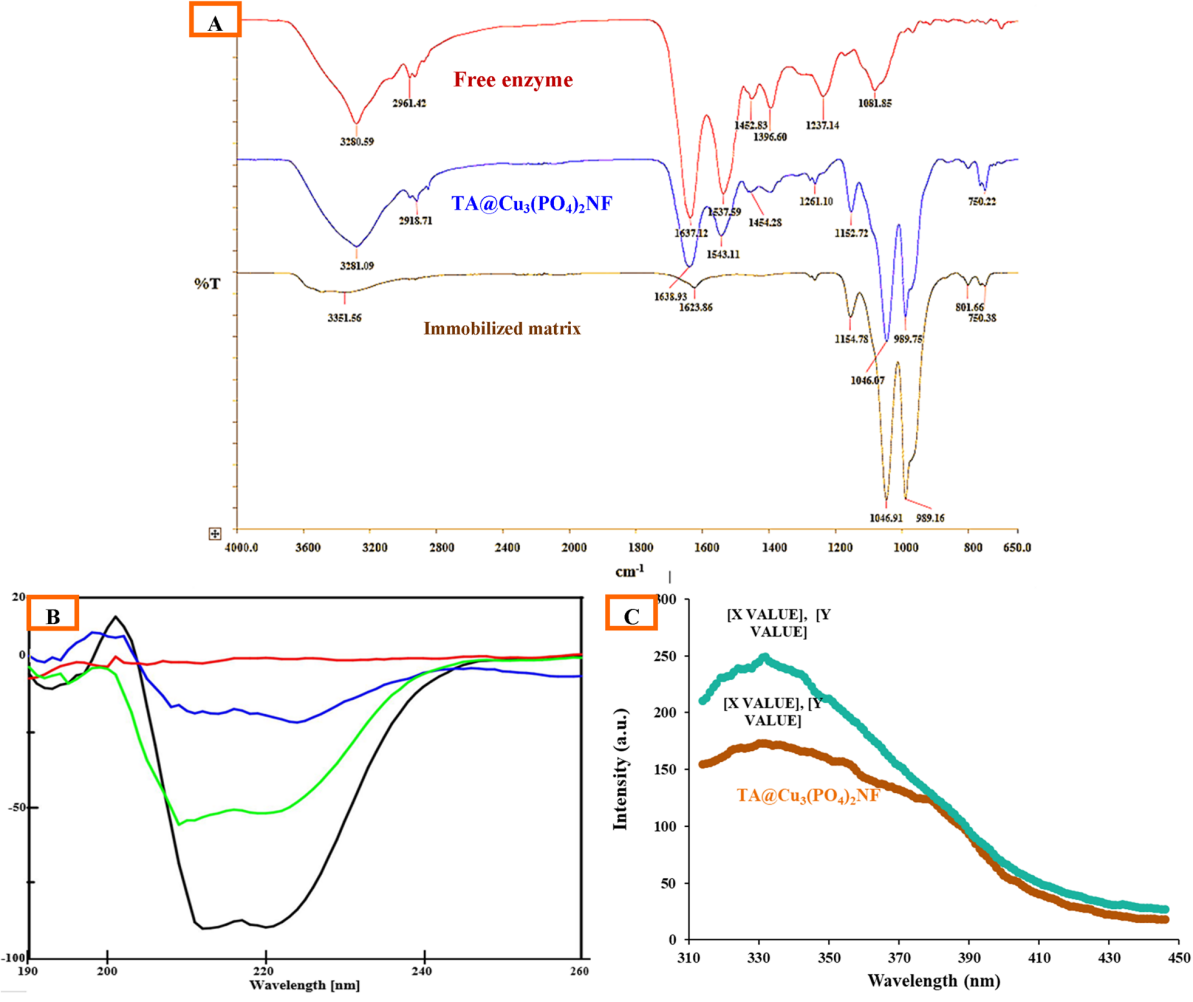
**A** Chromatogram of (*RS*)-α-MBA; **B** chromatogram of (*S*)-α-MBA; **C** chromatogram for catalytic conversion of (*R*)-α-MBA into acetophenone at different time periods

The FTIR spectra of the immobilized matrix and the free and immobilized enzyme were scanned in the region of 650–4000 cm^−1^to confirm the presence of transaminase in the NFs (Fig. 5A). The immobilized matrix displayed the peak at 1154 cm^−1^, and 1046 cm^−1^and 989 cm^−1^ were recognized as the Cu–OH bending vibrations, the asymmetric and symmetric stretching vibrations of PO4^3−^, respectively (He et al. 2015). The amide I and II bands of the transaminase were observed at 1637 cm^−1^, derived mainly from the C = O stretching vibrations of the peptide linkages, and 1537 cm^−1^was primarily attributed to the in-plane NH bending vibration and CN stretching vibration in the transaminase (Wu et al. 2017). TA@Cu_3_(PO_4_)_2_NF exhibited peaks of the immobilized matrix and the free enzyme validated the presence of proteins in the NFs. The hybrid nanoflower spectrum did not show any new absorption peaks or significant peak shifts in comparison to the immobilized matrices and free enzyme. These results indicated the enzyme immobilization was through self-assembly in hybrid NFs, instead of covalent conjugation.

#### Circular dichroism spectroscopy

Circular dichroism spectroscopy in a distant UV wavelength range (260–170) covers peptide bond absorption, and can be used to characterize and quantify secondary structural features such as, α-helical, β-strand, and an unordered structure (Miles and Wallace 2015). The reaction mixture without the enzyme (immobilized matrix) had no contribution to the CD spectrum. Figure 5B depicted the CD spectrum of TA@Cu_3_(PO_4_)_2_ and free transaminase. From Table 2, it was clear that there were no significant changes in the secondary structure fraction ratio of the immobilized enzyme as compared to the free transaminase. These results demonstrated the preservation of secondary enzyme structure inside the immobilized matrix.

**Table 2:** Secondary structure fraction ratio of free and immobilized enzyme

Secondary structure	Fraction ratio (%)	
	Free enzyme	Immobilized enzyme
Helix	28.3	30.8
Beta	0.0	0.0
Turn	35.1	36.3
Random	36.5	32.9
Total	100.0	100.0

#### Fluorescence study

The fluorescence spectra of the free enzyme solution exhibited emission maxima (*λ*_em_) at 332 nm, with an intensity of 248.69 arbitrary units, and TA@Cu_3_(PO_4_)_2_ NF exhibited an emission maxima at 330 nm, with an intensity of 172.75 arbitrary units (Fig. 5C). TA@Cu_3_(PO_4_)_2_NF did not show any notable *λ*em shift, which showed a preservation of the tertiary enzyme structure inside the immobilized matrix. The quenching effect was observed due to the Cu (II) metal ion (Plotnikova et al. 2016).

### Application of TA@Cu_3_(PO_4_)_2_NF in the resolution of (RS)-α-methyl benzyl amine

**Fig. 6:**
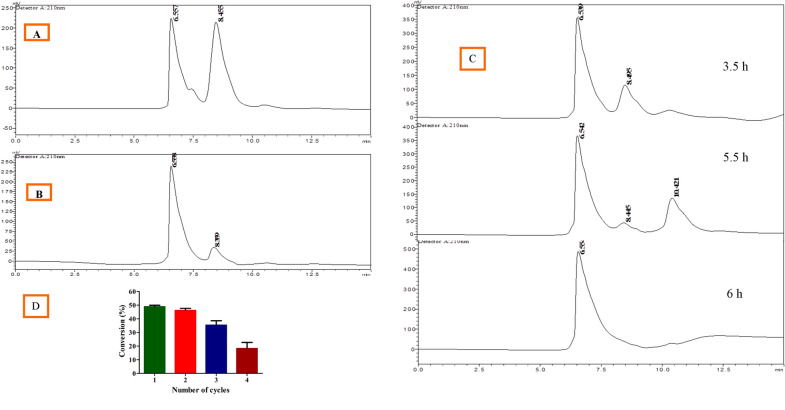
**A** FTIR spectra overlay of free enzyme, immobilized enzyme [TA@Cu3(PO4)2NF], and immobilized matrix. **B** CD spectra overlay of free enzyme, TA@Cu_3_(PO_4_)_2_NF, sonicated enzyme, and immobilized matrix. **C** The overlay of fluorescence spectra: free enzyme and TA@Cu_3_(PO_4_)_2_NF. **D** reusability study of TA@Cu_3_(PO_4_)_2_NF

Reusability is a significant factor in the industrial application of enzymes. The reusability study of developed hybrid NFs [TA@Cu_3_(PO_4_)_2_NF] was carried out through the kinetic resolution of the (*RS)*-α-methyl benzyl amine (Fig. 6A; Table 3). The kinetic resolution of the racemic mixture of α-MBA by TA caused (R)-α-MBA deamination and (S)-α-MBA remained without any structural changes (Fig. 6B; Table 4). The enantioselective catalytic conversion of (*R*)-α-MBA into acetophenone at different time periods (3.5, 5.5, and 6.0 h) is summarized in Table 5. The enzymatic resolution by TA@Cu_3_(PO_4_)_2_NF resulted in 49.93% conversion of (R)-α-MBA to acetophenone with 99.85% enantiomeric excess after 6.0 h of reaction (Fig. 6C). The TA@Cu_3_(PO_4_)_2_NF was consecutively used in four cycles for the catalytic conversion of (*R*)-α-MBA into acetophenone (Fig. 6D). As shown in Fig. 6D, in the initial two cycles, the percentage conversion of substrate into product was more than 47% and later it decreased to 18%. A decrease in substrate conversion might be due to the loss of nanoflower preparation during the recycling or washing process. It may be due to the leaching of the enzyme from the nano-preparation, however, it retained up to 37% of relative activity after four cycles of reuse. The reusability can be improved by adding some stabilizer during nanoflower synthesis which is known to reduce leaching of enzymes and also by the careful washings of reaction mixture during recycling. These results are in consistent with previous studies carried out by other researchers in which they immobilized different enzymes for the kinetic resolution of the racemic mixture (Rai et al. 2018; Soni et al. 2018; Dwivedee et al. 2018).

**Table 3:** HPLC data of (*RS*)-α-methyl benzyl amine before catalysed by (*R*)-specific transaminase

Peak	Retention time	Area	Height	Area %	Height %
1	6.557	6,800,329	2,20,596	46.461	52.336
2	8.455	7,836,380	2,00,900	53.539	47.664
Total		14,636,709	4,21,496	100.00	100.00

**Table 4:** HPLC data of (*RS*)-α-methyl benzyl amine after catalysed by (*R*)-specific transaminase

Peak	Retention time	Area	Height	Area %	Height %
1	6.558	7,496,306	2,42,475	89.210	88.375
2	8.359	9,06,656	31,896	10.790	11.625
Total		8,402,963	2,74,370	100.00	100.00

**Table 5:** Catalytic conversion of (*R*)-α-MBA into acetophenone at different time periods

Time (h)	Conversion (%)	ee (%)
3.5	28.31	53.55
5.5	44.43	88.08
6	49.93	99.85

## Conclusion

In this study, we established a novel method of transaminase copper phosphate nanoflowers synthesis. Transaminase copper hybrid nanoflowers were basically synthesized by sonication for 20 min at room temperature. It produced hierarchically designed flower-like morphology with greater stability and enzyme activity. The effects of all the reaction parameters (such as, sonication time, amplitude, buffer pH, enzyme, and metal salt concentration) on the morphology of the NFs and TA activity were systematically investigated and optimized. The resultant hybrid NFs exhibited higher reusability up to four cycles, with a retention of 37% activity. Additionally, TA@Cu_3_(PO_4_)_2_NF was applied in the kinetic resolution of (*RS*)-α-methyl benzyl amine. Moreover, this developed method could be applied to promptly synthesize NFs for numerous applications in enzyme catalysis, biofuel cells, and biosensors, and would magnify the exploitation of NFs in the various fields of biotechnology.

## Data Availability

All data generated or analysed during this study are included in this published article.
